# Detectable Bias between Vascular Ultrasound Echo-Tracking Systems: Relevance Depends on Application

**DOI:** 10.3390/jcm12010069

**Published:** 2022-12-21

**Authors:** Afrah E. F. Malik, Alessandro Giudici, Koen W. F. van der Laan, Jos Op ’t Roodt, Werner H. Mess, Tammo Delhaas, Bart Spronck, Koen D. Reesink

**Affiliations:** 1 Department of Biomedical Engineering, CARIM School for Cardiovascular Diseases, Maastricht University, Universiteitssingel 50, 6229 ER Maastricht, The Netherlands; 2 GROW School for Oncology and Reproduction, Maastricht University, Universiteitssingel 50, 6229 ER Maastricht, The Netherlands; 3 Department of Internal Medicine, CARIM School for Cardiovascular Diseases, Maastricht University, Universiteitssingel 50, 6229 ER Maastricht, The Netherlands; 4 Department of Clinical Neurophysiology, CARIM School for Cardiovascular Diseases, Maastricht University, Universiteitssingel 50, 6229 ER Maastricht, The Netherlands; 5 Macquarie Medical School, Faculty of Medicine, Health and Human Sciences, Macquarie University, 75 Talavera Rd., Sydney, NSW 2109, Australia

**Keywords:** echo-tracking, vascular ultrasound, arterial properties, arterial stiffness

## Abstract

The Esaote MyLab70 ultrasound system has been extensively used to evaluate arterial properties. Since it is reaching end-of-service-life, ongoing studies are forced to seek an alternative, with some opting for the Esaote MyLabOne. Biases might exist between the two systems, which, if uncorrected, could potentially lead to the misinterpretation of results. This study aims to evaluate a potential bias between the two devices. Moreover, by comparing two identical MyLabOne systems, this study also aims to investigate whether biases estimated between the MyLabOne and MyLab70 employed in this study could be generalized to any other pair of similar scanners. Using a phantom set-up, we performed *n* = 60 measurements to compare MyLab70 to MyLabOne and *n* = 40 measurements to compare the two MyLabOne systems. Comparisons were performed to measure diameter, wall thickness, and distension. Both comparisons led to significant biases for the diameter (relative bias: −0.27% and −0.30% for the inter- and intra-scanner model, respectively, *p* < 0.05) and wall thickness (relative bias: 0.38% and −1.23% for inter- and intra-scanner model, respectively *p* < 0.05), but not for distension (relative bias: 0.48% and −0.12% for inter- and intra-scanner model, respectively, *p* > 0.05). The biases estimated here cannot be generalized to any other pair of similar scanners. Therefore, longitudinal studies with large sample sizes switching between scanners should perform a preliminary comparison to evaluate potential biases between their devices. Furthermore, caution is warranted when using biases reported in similar comparative studies. Further work should evaluate the presence and relevance of similar biases in human data.

## 1. Introduction

Arterial properties, such as diameter, wall thickness, and distension, are extensively investigated in the literature [1,2,3,4,5,6], considering the valuable information they provide about cardiovascular health. Moreover, they are used to quantify arterial stiffness: an independent predictor of cardiovascular diseases [7]. Local arterial stiffness can be characterized by the distensibility coefficient and Young’s elastic modulus, among other indices. These indices require the assessment of the instantaneous diameter change and wall thickness by means of ultrasound echo-tracking [8,9,10].

Efforts made by prof. Hoeks and his group [2,11,12] represent seminal endeavors for the utilisation of ultrasound echo-tracking in the field of large artery (patho-)physiology. Their efforts mainly focused on analysing radiofrequency (RF) signals to estimate arterial distensibility. Hoeks’ group developed the necessary software and hardware (ART.LAB) to be integrated ultimately with the MyLab70 (Esaote Europe B.V. Maastricht, the Netherlands) and to present a top-class research-oriented echo-tracking system. In the past two decades, the MyLab70/ART.LAB combo has been used extensively in expert centers to quantify arterial elastic and geometrical properties, predominantly in a research context [13,14,15]. At the same time, ultrasound manufacturers continued to incorporate such technology into commercial devices with the objective of bringing the technology to clinical practice. Today, the MyLab70/ART.LAB has reached its end-of-service-life, forcing ongoing longitudinal epidemiological and interventional studies to switch to another scanner, which, because of technical differences, may not necessarily provide identical results. For instance, the Maastricht Study opted for the MyLabOne with RFQAS and RFIMT functionalities, plus a radiofrequency output license (Esaote) [14], with technology based on the original radiofrequency tracking [2]. The newer system represents a portable, integrated, and more affordable substitute to MyLab70/ART.LAB. The two systems, however, have different technical characteristics, and hence, their use at two-time points of a longitudinal study might result in a bias in the follow-up measurement. Not considering or correcting for such an ultrasound system-related bias may lead to misinterpretation of results and, thereby, erroneous conclusions.

The primary aim of the present study was to compare MyLabOne- and MyLab70-based echo-tracking systems to assess potential bias in quantifying diameter, wall thickness, and distension. In addition, we explored if such biases might also arise when comparing two MyLabOne systems with identical specifications. We conducted the comparative measurements in two steps, using a phantom set-up and three ultrasound systems: MyLabOne I, MyLab70, and MyLabOne II. First, we compared MyLab70 and MyLabOne I. We refer to this comparison as inter-scanner model comparison. Next, we estimated the biases between MyLabOne I and MyLabOne II. We refer to this comparison as intra-scanner model comparison. To confirm that the inter- and intra-system model biases estimated in this study are not spurious but that they originate from real intra- and inter-system model differences, we also performed an intra-device comparison. To this end, we assessed the differences between two measurement sets performed with MyLabOne I.

## 2. Materials and Methods

### 2.1. Ultrasound Scanners

Measurements in this study were performed using three different ultrasound systems: MyLabOne I, MyLabOne II, and MyLab70. A summary of the specifications of these systems is shown in Table 1. MyLab70 was equipped with a linear array transducer operating at 7.5 MHz and had a practical axial resolution of 0.125 mm. MyLabOne systems were equipped with linear array transducers operating at 10 MHz. The systems had a practical axial resolution of approximately 0.120 mm. All three systems were operating in fast B-mode, with high frame rates of 498 and 524 for MyLab70 and the MyLabOne systems, respectively. These high frame rates are achieved by generating multiple M-lines separated by 0.98 mm in the longitudinal direction of the probe. The number of M-lines is, however, different between the two scanners: *n* = 19 for MyLab70 and *n* = 14 for MyLabOne. In addition, all three scanners enabled the recording of raw radiofrequency signals sampled at 50 MHz for MyLab70 and 33 MHz for MyLabOne.

### 2.2. Phantom Configuration

**Figure 1:**
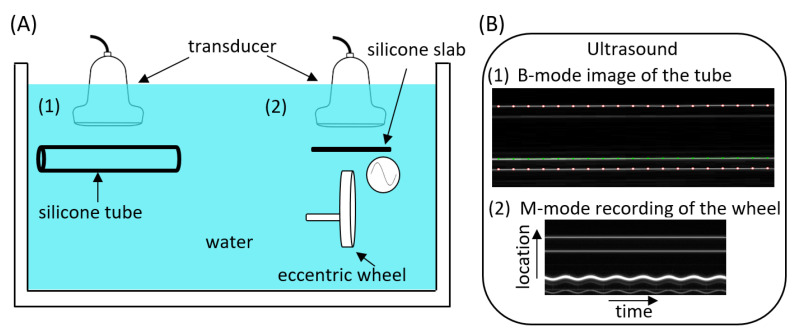
Study set-up for ultrasound system bias estimation (**A**) and how it appeared in the ultrasound measurements (**B**). Two-part phantom set-up consisting of a silicone tube (1), used to assess inter- and intra-scanner biases in diameter and wall thickness, and an eccentric wheel with a silicone slab mounted on top of it (2), used to assess the bias in distension. (B.1): Example of a B-mode image for the silicone tube. The white dot markers indicate the outer tube–water echo interfaces (appearing as horizontal white lines) of the near (top) and far (bottom) walls of the silicone tube used to measure the diameter. The green line indicates the inner tube–water echo interface of the far wall, which, together with the far wall outer echo interface, was used to measure wall thickness. (B.2): Example of an M-mode acquisition of the wheel ‘distension’. The white sinusoidal line reflects the motion of the wheel surface, while the two less echogenic parallel reflections above the undulating line indicate the silicone slab.

To perform the inter- and intra-ultrasound system model comparisons to estimate scanner biases for assessing diameter, wall thickness, and distension, a two-part phantom set-up was used (Figure 1). The first part consisted of a static silicone tube with an outer diameter of 12.4 mm and a wall thickness of 1 mm. This part was used to estimate biases between the ultrasound system pairs for diameter and wall thickness. The second part consisted of an eccentric wheel connected to a motor via a rod, which was inserted in the wheel 300 μm off centre. Thus, measuring the instantaneous location of the top surface of the wheel results in a sinusoidal distension waveform with a peak-to-peak amplitude of 600 μm. A silicone slab was mounted above the wheel to mimic the artery near wall. This near wall was held in a fixed position; hence, it did not contribute to the simulated vessel distension. The phantom set-up and the transducer lens were immersed in tap water at room temperature to enable ultrasound propagation.

### 2.3. Data Acquisition

To compare MyLabOne I and MyLab70, we obtained *n* = 60 repeated RF acquisitions for both scanners. Post hoc analysis using data from the previously mentioned comparison revealed that after about 40 repeated measurements, the bias and confidence intervals (CI) remained relatively constant for all examined variables indicating that *n* = 40 repeated measurements were sufficient to provide a reliable estimate of the bias. Therefore, for the intra-device model comparison, we performed *n* = 40 repeated measurements using MyLabOne I and MyLabOne II for diameter and wall thickness. Moreover, we inferred from the inter-device comparison that part of the distension recordings might be lost potentially due to uncontrolled saturation; hence, *n* = 60 repeated distension measurements were performed for the intra-device model case. The MyLabOne I measurement set available for the inter-scanner model comparison was also used to perform an intra-device comparison. For all the studied variables, two groups were created by dividing this measurement set into two groups based on the order of performing the measurement (even and odd). Individual acquisitions were performed by repositioning the ultrasound probe at random distances (ranging between 1 and 3 cm) from the tube and the wheel. This was conducted to simulate in vivo situations due to the fact that the depth from the skin to the carotid artery varies between different individuals.

### 2.4. Data Processing

The diameter was defined as the distance between the near and far wall outer silicone-water reflections and is indicated by white dotted lines in Figure 1B.1. Wall thickness was defined as the distance in the far wall between the inner and outer silicone-water reflections. Both diameter and wall thickness were estimated based on longitudinal acquisitions covering 19 and 14 equidistant M-lines for Mylab70 and MyLabOne, respectively. On the contrary, cross-sectional acquisitions of the wheel motion were performed to estimate distension. Since the wheel thickness was 4 mm and the distance between the ultrasound M-lines was approximately 1 mm, only a few M-lines covered the wheel. Therefore, we estimated the distension based on a single M-line with the most wheel coverage [9]. This was deduced based on the brightness of the corresponding B-mode of the line, indicating a strong wheel reflection. RF signals were processed in MATLAB (MATLAB R2020b; The MathWorks, Natick, MA, USA) using proprietary wall-tracking software that was previously described in [2,12,16].

### 2.5. Statistical Analyses

RF recordings were acquired for at least five seconds for all scanners. Biases were quantified as means ± 95% CI and are reported in absolute and relative terms. For all three considered variables, the absolute bias was calculated as the difference between the average of estimates obtained with MyLabOne I and MyLab70 (i.e., MyLabOne I- minus MyLab70-derived values) and between those obtained with MyLabOne I and MyLabOne II (i.e., MyLabOne I- minus MyLabOne II-derived values) for the inter-device and intra-device comparisons, respectively, and tested with an independent sample Student’s *t*-test. The relative bias was defined as the absolute bias normalized with respect to the mean value of both systems. Precision was assessed by the estimates’ standard deviation (SD) and compared with F-test. Statistical analyses were performed using SPSS version 27 (SPSS, Chicago, IL, USA). A two-sided *p*-value <0.05 was considered statistically significant.

## 3. Results

### 3.1. MyLabOne I vs. MyLab70

Post hoc, 21 MyLabOne I distension recordings were excluded due to the uncontrolled saturation in the corresponding RF complex [17].

**Figure 2:**
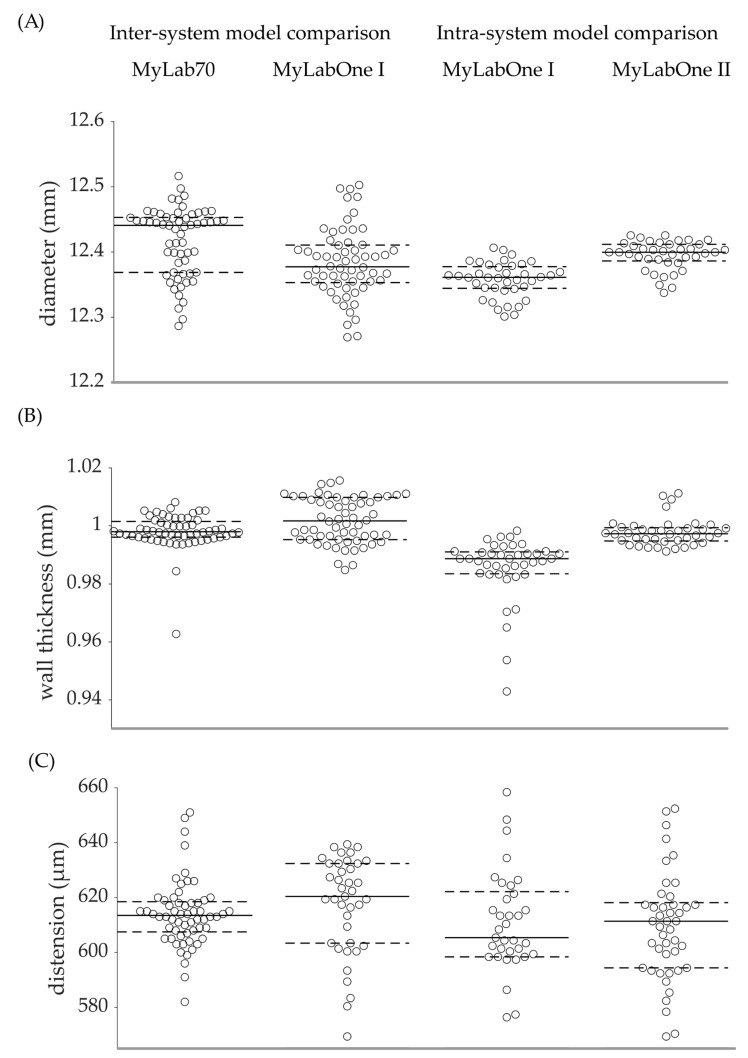
Overview of absolute values of all repeated measurements performed for the inter- and intra-scanner model comparisons. Measurements were performed with MyLab70, MyLabOne I, and MyLabOne II for diameter (**A**), wall thickness (**B**), and distension (**C**). Solid lines indicate the medians and dashed lines indicate the 25th and 75th percentiles.

The diameter obtained with MyLabOne I was significantly lower than that obtained with MyLab70 (12.3830 vs. 12.4170 mm, *p* = 0.001), corresponding to a relative bias of −0.27%. However, the precision of the diameter measurements defined as the SD was similar for the two scanners (0.0533 vs. 0.0527 mm, *p* = 0.542). Compared to MyLab70, MyLabOne I resulted in a significantly higher wall thickness (1.0019 vs. 0.9981 mm, *p* = 0.004) which translated into a relative bias of 0.38%. Further, MyLabOne I yielded a significantly higher standard deviation for wall thickness (0.0079 vs. 0.0062 mm, *p* < 0.001). The SD obtained with MyLabOne I for distension was significantly higher than that achieved with MyLab70 (17.8 vs. 12.1 μm, *p* = 0.047). However, we found no significant difference between the two scanners for distension (617.0 vs. 614.1 μm, *p* = 0.333) (Table 2 and Figure 2).

### 3.2. MyLabOne I vs. MyLabOne II

Of the distension measurements performed for the intra-scanner model comparison, *n* = 23 were excluded for MyLabOne I and *n* = 15 were excluded for MyLabOne II due to uncontrolled saturation in the corresponding RF complex.

The comparison of the two MyLabOne systems yielded significantly different diameter estimates (12.3569 vs. 12.3945 mm, *p* < 0.001), corresponding to a relative bias of −0.30%. However, the two systems yielded similar SDs (0.0222 vs. 0.0267 mm, *p* = 0.343) for diameter. The two systems resulted in significantly different wall thickness measurements (0.9855 vs. 0.9976 mm, *p* < 0.001), translating into a relative bias of −1.23%. Further, the two scanners yielded significantly different SDs of the wall thickness (0.0110 vs. 0.0048 mm, *p* = 0.013). We found no significant difference between the distension estimates obtained with the two scanners (609.5 vs. 610.2 μm, *p* = 0.892). Similarly, there was no significant difference between the SDs of the distension estimates obtained with the two systems (25.5 vs. 21.4 µm, *p* = 0.591) (Table 2 and Figure 2).

### 3.3. MyLabOne I vs. MyLabOne I

The results of this comparison are shown in Table 2. The intra-device comparison yielded statistically non-significant differences for diameter (difference −0.0012 mm, *p* = 0.929), wall thickness (difference = −0.0026 mm, *p* = 0.198), and distension (difference −3.6 μm, *p* = 0.529). Similarly, the two measurement sets of MyLabOne I resulted in statistically non-significant SDs for all the examined variables (*p* > 0.05). Because of the considerable intercurrent time between the two available MyLabOne I measurement sets (i.e., one set for the inter- and one for the intra- system model comparisons) as well as the lack of consistency in measurement conditions/set-up status, we refrained from intra-device comparison based on the two available MyLabOne I measurement sets.

## 4. Discussion

Using a phantom set-up, this study assessed the inter- and intra-scanner biases between MyLabOne I- and MyLab70-based echo-tracking systems for measuring arterial diameter, wall thickness, and distension. Our results show detectable biases for diameter and wall thickness but not for distension. This held true for the comparison between the MyLab70 and a MyLabOne I system, as well as for the comparison between two MyLabOne systems. Biases were in the same order of magnitude in both comparisons. All biases were very small with respect to the values reported in the literature for studies comparing two echo-tracking systems (Table 3) [1,3,4]. Based on our results, research studies should adhere to one device unless switching is necessary. Whenever replacement is unavoidable, a comparison between the two systems should be performed to establish the amount of bias, even if the devices have the same vendor and model.

To the best of our knowledge, this is the first study to compare MyLabOne and MyLab70, as well as two MyLabOne systems with identical specifications. MyLab70 and MyLabOne share several common features. Indeed, they are both RF-based echo-tracking systems designed and manufactured by the same manufacturer. In addition, the two systems employ conceptually similar RF tracking approaches [2]. As shown in Table 3, the biases between MyLabOne and MyLab70 for all the examined variables were lower than the values reported by similar studies comparing two different devices/models. This indicates that MyLabOne is a good substitute for MyLab70.

To ensure that the non-significant bias found in the case of the distension was not due to insufficient statistical power, we performed a post hoc power analysis. This analysis was performed using G*Power version 3.1.9.4: an open-source statistical power analysis tool available at https://www.gpower.hhu.de (accessed on 23 June 2021) [18]. The power analysis showed that a sample size of 39 would enable us to detect an effect size greater than 0.64 (power 80% and a two-sided α = 0.05). Note that based on our study design, we would, thus, be able to detect a bias greater than 64% of the device’s precision.

Biases between MyLabOne I and MyLab70 systems for all examined variables did not exceed 0.5%, which appears clinically irrelevant for personalized risk stratification and diagnosis (e.g., in the context of cardiovascular risk assessment). However, the findings presented in this study may have direct implications for research studies, particularly follow-up designs. By alleviating the effect of device-related biases on the outcomes of these studies, the findings presented here have an indirect clinical relevance. For such studies, an appraisal of the relevance of the bias depends on multiple factors, with the sample size/statistical power being the most important. For instance, for the same value of the bias, a low population variability would lead to significant results with a small sample size, while larger variability requires a larger sample size for the results to be relevant.

**Figure 3:**
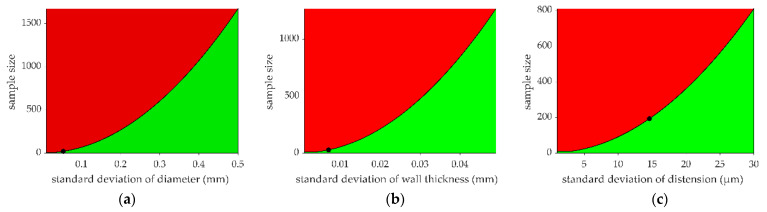
Lowest sample size (*n*_min_) for estimated biases to be considered relevant. *n*_min_ is estimated using several values of standard deviation for (**a**) diameter, (**b**) wall thickness, and (**c**) distension. We assumed that variability within research studies conducted in humans could be expected to be larger than that observed here using a phantom and, hence, considered a range of variabilities (defined with SD) in quantifying *n*_min_. The black line represents *n*_min_ as a function of SD, while the red area represents sample sizes for which estimated biases are considered significant. Black dots represent *n*_min_ for a significant bias calculated using the SDs observed in this study, and they correspond to 21, 28, and 193 samples for diameter (**a**), wall thickness (**b**), and distension (**c**), respectively.

Let one consider the lowest sample size (*n*_min_) beyond which the estimated biases would be considered relevant. In other words, studies with a sample size exceeding *n*_min_ should consider the effect of the inter-/intra-scanner bias in their analysis and interpretation. Figure 3 shows the results of our calculations of *n*_min_ for a range of population variabilities. Based on the results presented in Figure 3, research studies are recommended to consider their population variability/statistical power when evaluating the relevance of the biases detectable between MyLabOne and MyLab70 systems.

This study found differences between two identical-on-paper systems (MyLabOne I and MyLabOne II) for diameter, and wall thickness, indicating that the results found here for the inter-scanner comparison could not be generalized to any other pair of similar scanners. These findings also imply that caution is warranted when using systematic biases reported in similar comparative studies. A potential explanation for the intra-scanner model differences relates to the different operational periods between our two systems and the supposed ‘wear’ effect on data quality. Another possible explanation relates to the uncertainty in the manufacturing process, which is determined by the adopted tolerance, and the admissible variation in the end product.

The intra-device comparison was performed to check if the differences found in the cases of the inter- and intra- system model comparisons were spurious, originating from factors such as study set-up, environmental conditions, and wear effect or if they were real, originating from inter- and intra- system model differences. Intra-device differences for all the studied variables were not statistically significant (Table 2), confirming that the significant differences found in the cases of inter- and intra-scanner model comparisons originated from real device/device model differences. Compared to inter- and intra- system model differences, intra-device differences were smaller for diameter and wall thickness and larger for distension. We believe that the difference found in the case of distension was caused by the relative uncertainty of the distension estimate.

For studies switching between devices, a similar phantom set-up and approach could be used to calibrate the new system against the old one to avoid any effect that a systematic bias between the two systems could have on the study outcomes. Phantom set-ups are controllable and provide repeatable estimates; hence, they are superior to human data for calibration purposes. By using a phantom, one mitigates additional uncertainty in bias estimates originating from human data variability.

This study has several possible limitations: (1) Some distension recordings were excluded due to saturation in the RF complex. This problem was experienced with the MyLabOne systems but not with MyLab70. The eccentric wheel used in the phantom set-up was made of a strong reflector; hence, with certain gain settings, the peaks of the incoming RF signal may exceed the dynamic range (16 bit or 96 dB) of the scanner. While adjusting the gain setting was possible with MyLab70 during the RF acquisitions, this option was not available for MyLabOne, explaining the occurrence of saturation issues. (2) The set-up used here consisted of homogeneous materials, and diameter and wall thickness measurements were performed under static conditions. Tissue inhomogeneity and vessel wall motion might alter bias estimates under in vivo situations. (3) In vivo arterial diameter and wall thickness (defined as intima-media thickness) are typically smaller than those of the phantom tube. Hence, the effect of the ultrasound scanners’ limited axial resolution may be expected to be more pronounced during in vivo settings.

## 5. Conclusions

The present study found detectable inter- and intra-scanner model biases for diameter and wall thickness measurements but not for distension measurements. The existence of a detectable bias between two identical systems/models indicates that the biases estimated in the present study cannot be generalized to any other pair of scanners. Therefore, studies with large sample sizes and particularly those with longitudinal designs, in which a change in or an exchange of scanners is necessary, should check for the presence of biases between devices following our approach. Further work should evaluate the presence and relevance of biases in (existing) human studies.

## Figures and Tables

**Table 1:** Specifications of echo-tracking systems used in the study.

	MyLab70	MyLabOne I & II
Operating frequency (MHz)	7.5	10
RF sampling frequency (MHz)	50	33
Frame rate (fps)	498	524
No. of M-lines	19	14
Practical * axial resolution (mm)	0.125	0.120
Approx. cost	120 k	25 k
RF wall tracking	ART.LAB	RF module
RF output format	.r70	.zrf

RF—radiofrequency; * Practical here refers to the resolution estimated and based on the actual bandwidth measured from received RF signals in contrast to theoretical axial resolution.

**Table 2:** Diameter, wall thickness, and distension as determined by MyLabOne I, MyLab70, and MyLabOne II for the inter- and intra-device model and the intra-device comparisons.

**Inter-system model comparison**						
				**Bias (95% CI)**		***p***
				**Absolute**	**Relative (%)**	
		**MyLabOne I**	**MyLab70**			
Diameter (mm)	*n*	60	60			
	Mean	12.3830	12.4170	−0.0339 (−0.0530 to −0.0147)	−0.27 (−0.43 to −0.12)	0.001
	SD	0.0533	0.0527			0.542
Wall thickness (mm)	*n*	60	60			
	Mean	1.0019	0.9981	0.0038 (0.0013 to 0.0064)	0.38 (0.13 to 0.63)	0.004
	SD	0.0079	0.0062			<0.001
Distension (µm)	*n*	39	60			
	Mean	617.0	614.1	2.9 (−3.0 to 8.8)	0.48 (−0.56 to 1.51)	0.333
	SD	17.8	12.1			0.047
**Intra-system model comparison**						
		**MyLabOne I**	**MyLabOne II**			
Diameter (mm)	*n*	40	40			
	Mean	12.3569	12.3945	−0.0376 (−0.0484 to −0.0268)	−0.30 (−0.39 to −0.22)	<0.001
	SD	0.0222	0.0267			0.343
Wall thickness (mm)	*n*	40	40			
	Mean	0.9855	0.9976	−0.0121 (−0.0159 to −0.0084)	−1.23 (−1.60 to −0.85)	<0.001
	SD	0.0110	0.0048			0.013
Distension (µm)	*n*	37	45			
	Mean	609.5	610.2	−0.7 (−11.0 to 9.6)	−0.12 (−1.81 to 1.52)	0.892
	SD	25.5	21.4			0.591
**Intra-device comparison**						
		**MyLabOne I** **1st set**	**MyLabOne I** **2nd set**			
Diameter (mm)	*n*	30	30			
	Mean	12.3824	12.3837	−0.0012 (−0.0284 to 0.0260)	−0.01 (−0.23 to 0.21)	0.929
	SD	0.0500	0.0573			0.257
Wall thickness (mm)	*n*	30	30			
	Mean	1.0006	1.0032	−0.0026 (−0.0066 to 0.0013)	−0.26 (−0.66 to 0.13)	0.198
	SD	0.0086	0.0070			0.364
Distension (µm)	*n*	20	19			
	Mean	615.2	618.8	−3.6 (−14.8 to 7.6)	−0.59 (−2.41 to 1.23)	0.529
	SD	18.9	16.8			0.697

SD—standard deviation; CI—confidence intervals.

**Table 3:** Studies found in the literature that compare two different ultrasound devices for measuring arterial diameter, wall thickness, and distension.

Variable	Study	Type of Data	Compared Devices	*n*	Absolute Bias	Relative Bias (%)
Diameter (mm)	Bozec et al., 2020 [1]	Carotid artery	Wall tracking system (WTS) and ART.LAB	188	0.119	1.8
	Palombo et al., 2012 [4]	Carotid artery	Two RF-based systems	105	0.263	3.4
	Morganti et al., 2005 [3]	Carotid artery	Multigate Doppler system against commercially available ultrasound device	37	0.05	0.7
	**This study, 2022**	**Phantom set-up**	**Esaote MyLabOne I and MyLab70**	**60**	**0.0339**	**0.27**
Wall thickness (mm)	Bozec et al., 2020 [1]	Carotid artery	WTS and ART.LAB	186	0.046	6.1
	**This study, 2022**	**Phantom set-up**	**Esaote MyLabOne I and MyLab70**	**60**	**0.0038**	**0.38**
Distension (µm)	Bozec et al., 2020 [1]	Carotid artery	WTS and ART.LAB	181	23	4.3
	Palombo et al., 2012 [4]	Carotid artery	Two RF-based systems	105	91	22
	Morganti et al., 2005 [3]	Carotid artery	Multigate Doppler system against commercially available ultrasound device	37	34	6.8
	**This study, 2022**	**Phantom set-up**	**Esaote MyLabOne I and MyLab70**	**39 and 60**	**2.9**	**0.48**

Bold texts highlight the current study.

## Data Availability

Data will be made available upon request to the corresponding author.
